# Omission

**Published:** 1882-09-20

**Authors:** 


### EDITORIALS AND MISCELLANEOUS.

OMISSION.—We regret the accidental omission, by our printers, of a part of the interesting paper in our last issue entitled "*Clinical Report of a Case of Compound Comminuted Fracture*," *etc.*, by Drs. Hays Bros., of Marianna, Ark.

